# A spectrum of non-spore-forming fermentative and non-fermentative Gram-negative bacteria: multi-drug resistance, extended-spectrum beta-lactamase, and carbapenemase production

**DOI:** 10.3389/frabi.2023.1155005

**Published:** 2023-04-28

**Authors:** Yasin Desalegn, Adane Bitew, Amanuel Adane

**Affiliations:** ^1^ Addis Ababa Public Health Research and Emergency Management Directorate, Addis Ababa, Ethiopia; ^2^ Department of Medical Laboratory Science, College of Health Sciences, Addis Ababa University, Addis Ababa, Ethiopia; ^3^ Saint Peter’s Specialized Tuberculosis Referral Hospital, Addis Ababa, Addis Ababa Administrative Region, Ethiopia

**Keywords:** Gram-negative bacteria, MDR, ESBLs, carbapenemase, fermentative and non- fermentative

## Abstract

**Background:**

In developing countries, the co-existence of a high burden of infectious diseases caused by Gram-negative bacteria and the rapid increase and spread of multidrug-resistant bacteria have become a serious health threat.

**Objective:**

Profiling of Gram-negative bacteria and determining the magnitude of their antimicrobial resistance among patients.

**Results:**

A total of 175 non-spore-forming Gram-negative bacteria were isolated from 873 different clinical samples. Of a total of 175 bacteria, 154 (88%) were fermentative Gram-negative bacteria, while 21 (12%) were non-fermentative Gram-negative bacteria*. E. coli* with a frequency of 58.3% and *K. pneumoniae* with a frequency of 18.3% were the predominant fermentative Gram-negative bacteria, while *P. aeruginosa* 9 (5.1%) and *A. baumannii* 6 (3.4%) were the predominant non-fermentative Gram-negative bacteria. The highest percentage level of antibiotic resistance was seen against ampicillin (86%), and the lowest against meropenem (9.8). About 49 (28%) Gram-negative bacilli were positive for ESBLase. The overall prevalence rate of MDR bacteria was 80.5%, of which 100% of A. *baumannii*, 90.6% *of K. pneumonia.* Sixteen isolates were resistant to meropenem, out of which 11 tested for carbapenemase production. Five of the nine were metallo-lactamase producers, with the remaining four being serine carbapenemase producers.

**Conclusion:**

The prevalence of Gram-negative bacterial infection was found to be 20%, with a significant proportion (80.0%) due to fermentative Gram-negative bacteria and the remaining 20% due to non-fermentative Gram-negative bacteria. The study has also demonstrated a high prevalence rate of MDR, ESBLase, and carbapenemase-producing Gram-negative bacteria. Antimicrobial resistance of Gram-negative bacteria should be monitored on a regular basis, and an effective infection control program should be implemented.

## Introduction

Non-spore-forming Gram-negative bacilli (NGNB) have been classified as non-fermentative Gram-negative bacilli (NFGNB) or fermentative Gram-negative bacilli (FGNB). Non-spore forming Gram-negative bacteria are heterogeneous and composed of many medical important species, of which *Pseudomonas aeruginosa*, *Acinetobacter baumannii, Klebsiella pneumoniae, Enterobacter* species, *Escherichia coli, and Enterococcus faecium are* a few among the list. Most of the above-listed bacteria are members of the dangerous small group of pathogens called the ESKAPE bugs, which stand for *Enterococcus faecium, Staphylococcus aureus, K. pneumoniae, A. baumannii, P. aeruginosa, and Enterobacter* species (Rice, 2008). According to Rice, 2008, the ESKAPE bugs are extremely important bacteria as they account for the largest proportion of hospital-acquired infections, are more virulent, easily communicable, and develop resistance against most antibacterial drugs. The ubiquitous and intrinsic resistance characteristics of Gram-negative bacteria (GNB) to the commonly used antiseptics are responsible for their ability to occupy a wide range of hospital environments, including anesthesia equipment, sinks, intravenous fluids, and fomites, or the hands of medical staff, causing device-associated hospital infections (Gales et al., 2001; Mellmann et al., 2009; Kakati et al., 2015). Another feature of GNB is their relative ease of acquiring plasmid-containing genes that encode for Extended Spectrum β-Lactam Enzymes (ESBLase) and other resistance genes that confer resistance to many other classes of antibiotics (Brolund, 2014).

In developing countries, the co-existence of a high burden of infectious diseases caused by GNB and the rapid increase and spread of multidrug-resistant bacteria (MDR) have become a serious health threat as the latter limits the choice of appropriate treatment options (Gupta, 2008; Ayukekbong et al., 2017; Sharma et al., 2017; Yadav et al., 2020). Even though antimicrobial resistance occurs naturally over time, usually through gene or chromosomal mutations, misuse and overuse of antimicrobials have been identified as major factors in the development and spread of drug-resistant pathogens (World Health Organization, 2019). Furthermore, WHO, 2019 has declared that anti-microbial resistance is one of the top 10 global public health threats facing humankind.

Among the many drug classes, β-lactam antimicrobial agents have been the most commonly used to treat Gram-negative bacterial infection. GNB resistance to β-lactam antibiotics, including extended-spectrum penicillins, cephalosporins, monobactams, carbapenems, fluoroquinolones, and aminoglycosides, is one of the most serious problems confronting human health worldwide. The production of β-lactamase enzymes, particularly extended-spectrum β-lactamases that hydrolyze antibiotics with the β -lactam functional group, is the most important mechanism of resistance to β-lactam antibiotics. Given that ESBLase is plasmid mediated, it can easily be disseminated to members of the same or different species of GNB. Resistance to other antimicrobial classes, such as fluoroquinolones, aminoglycosides, and sulphonamides, has also been identified in extended-spectrum β-lactamases producing GNB (Schwaber et al., 2005; Chandel et al., 2011), rendering the most useful drugs ineffective and ultimately limiting treatment options for infection. This scenario leads to the wide use of carbapenems, which are often considered the last option for the treatment of infections related to MDR GNB isolates, which again leads to resistance to carbapenems (Tarashi et al., 2016; Farhan et al., 2019); those isolates are non-susceptible to at least one drug in three or more drug categories (Magiorakos et al., 2011). Yet, the use of carbapenems has led to the rapid selection of carbapenem-resistant GNB (Walsh, 2010), leaving colistin and tigecycline as the only options available for the treatment of ESBLase-producing GNB (Giamarellou, 2006; Morrill et al., 2015). Although bacterial antibiotic resistance develops naturally, its spread is primarily driven by overuse and misuse in healthcare systems, the environment, and agricultural activities (Roca et al., 2015). These factors, together with the lack and/or insufficiency of infection control activities, have worsened the situation (Ayukekbong et al., 2017). The poor regulatory system for antimicrobial agents over the counter and the supply of substandard antimicrobials are some of the other factors that facilitate the spread of drug-resistant bacteria (Osei-Safo et al., 2016; Sharma et al., 2017). Studies conducted in Africa, including Ethiopia, have demonstrated a mounting prevalence of Gram-negative bacteria resistant to commonly prescribed antibiotics (Kariuki and Dougan, 2014; Beyene et al., 2019; Tafese et al., 2020; Abdeta et al., 2021; Moges et al., 2021). Poor drug regulatory systems, a lack of established antibiotic stewardship, and a lack of laboratory testing capacities in Ethiopia dictate continuous surveillance studies on the level of drug resistance, the phenotypic profile of ESBLase, and carbapenemase producing GNB in different health settings. Assessing these factors in the local scenario is essential to knowing the epidemiology, the burden of disease, and antimicrobial resistance, as well as to designing and implementing appropriate infection control strategies in the health institutes that enable the reduction of the further occurrence and spread of resistant GNB in these institutions. Despite the untenable rate of antibiotic resistant bacterial infections reported in most Ethiopian health institutes, there is a substantial gap in the surveillance of these infections in several health institutes, especially in private health institutes where limited research has been done. Therefore, the objectives of this study are to determine the profile of NGNB and the magnitude of their antimicrobial resistance among patients attending Arsho Advanced Medical Laboratories, a private limited health institute.

## Materials and methods

### Study design, period, and study area

This prospective study was carried out between September 2018 and May 2019 in the Department of Microbiology and Molecular Biology at Arsho Advanced Medical Laboratory in Addis Ababa, the capital city of Ethiopia. Around 30-40 patients per day were sent to the department during our study period.

### Source of study population, sample size determination, and sampling procedure

The source of the study population was those patients referred to the Department of Microbiology and Molecular Biology at Arsho Advanced Medical Laboratory and bordering regions during the study period. The study populations were all patients seeking culture and susceptibility testing, excluding patients who were under drug treatment for less than two weeks.

### Sample size calculation

We have estimated the sample size using a single population proportion formula: n = Z^2^ P(1-P/d2), where

n = sample size; z = 95% level of confidence (1.96); P = population proportion of 50% (P = 0.5) as there are no studies conducted using the VITEK 2 compact system in our settings; d = margin of error (degree of accuracy desired) (d = 0.05). Hence, considering a 10% contingency, the minimum sample size was 422. However, in order to obtain a representative result, we raised the sample size to 873. Different clinical samples were collected from 873 patients by using a convenient sampling technique. Only one sample type per patient was collected. Laboratory request forms were used as a template to collect socio-demographic information from study participants. Prior to sample collection, however, written consent and assent were completed following the Helsinki declaration.

### Sample collection, processing, and culture

Clinical samples, including blood, urine, body fluids, nasal and ear swabs, were collected from ten sample collection sites. All samples were processed and inoculated onto blood agar (Oxoid, Basingstoke, Hampshire, UK), chocolate agar (Oxoid, Basingstoke, Hampshire, UK), MacConkey agar (Oxoid, Basingstoke, Hampshire, UK), and brain heart infusion blood culture bottles (Oxoid, Basingstoke, Hampshire) and incubated at an appropriate temperature for the appropriate period according to standard protocols related to each sample (Leber, 2016). The colony characteristics and Gram stain were used to characterize pure isolates of bacterial pathogens. Identification, antimicrobial susceptibility testing, and extended spectrum beta-lactamase production were performed with the VITEK 2 compact system using the AST-GN72 cards, in accordance with the manufacturer’s instructions.

#### Tests for carbapenemase production

Bacterial isolates that were not susceptible to meropenem (MEM 10µg) based on CLSI break points were subjected to confirmation for carbapenemase production (CLSI, 2018). Confirmation for carbapenemase production in Enterobacteriaceae and *P. aeruginosa* was done by the Modified Carbapenem Inactivation Method (mCIM). A second 2-ml tube of TSB was labeled for the EDTA-Modified Carbapenem Inactivation Method (eCIM) test to distinguish metallo -β-lactamase from serine carbapenemase in Enterobacteriaceae carbapenemase producers (CLSI, 2018).

#### Quality control

All laboratory assays were done by maintaining quality control procedures. Standard Operating Procedures (SOPs) were strictly followed, verifying that media met expiration dates and quality control parameters as per CLSI guidelines. Visual inspections of cracks in media or plates, unequal fill, hemolysis, evidence of freezing, bubbles, and contamination were performed. Culture media were tested for sterility and performance using reference strains of *E. coli* (ATCC 25922), and *P. aeruginosa* (ATCC 27853). The performance of VITEK 2-compact was also tested with *E. coli* (ATCC 25922) and *P. aeruginosa* (ATCC 27853). The performance of the equipment was monitored using standard procedures.

#### Data quality control

The data from the collection format was checked for its completeness, and test results were carefully recorded. The results of the culture, antibiotic susceptibility test, ESBL, and carbapenemase tests were documented carefully before entry into SPSS.

### Data analysis and interpretation

The data was entered and analyzed using Statistical Package for Social Sciences (SPSS) version 23.0. Descriptive statistics were computed and presented using figures and tables. To examine the relationship between dependent and independent variables, binary logistic regression was used. Moreover, a multivariate analysis was computed to identify factors that independently influence the occurrence of dependent variables. The odds ratio was used to show the strength of the association. P-values less than 0.05 were considered significant in all analyses.

## Results

### Distribution of clinical samples against gender and age

A total of 873 clinical samples were collected during the study period, of which 582 (66.7%) were collected from females and 291 (33.3%) from males. Among clinical samples, the highest number was 561 (64.2%), followed by blood at 107 (12.3%), and wounds at 91 (10.4%). The majority of clinical samples, 383 (43.9%), were collected from patients aged 25 to 44, with the lowest samples, 6 (0.7%), collected from children aged less than one year (**Table 1**).

**Table 1:** Distribution of clinical samples according to sex and age of study subjects (n=873).

Gender			Types of Sample n (%)							Total
			Urine	blood	Wound	CSF	Ear swab	Body fluids	Nasal swabs	
Male	Age	<1	2	0	0	0	0	0	0	2 (0.2)
		1-14	13	3	5	1	2	2	2	28 (3.2)
		15-24	6	4	3	3	2	1	2	21 (2.4)
		25-44	70	24	20	7	7	2	0	130 (14.9)
		45-64	27	14	5	5	5	3	0	59 (6.8)
		65	35	4	8	2	1	1	0	51 (5.8)
	Male Total		153 (17.5)	49 (5.6)	41 (4.7)	18 (2)	17 (1.9)	9 (1)	4 (0.5)	291 (33.3)
Female	Age	<1 1-14 15-24 25-44 45-64 65	1 37 32 180 88 70	1 4 11 28 7 7	1 10 4 23 8 4	1 7 5 11 4 4	0 2 5 6 4 0	0 1 1 5 1 3	0 4 2 0 0 0	4 (0.5) 65 (7.4) 60 (6.9) 253 (29) 112 (12.8) 88 (10)
	Female Total		408 (46.7)	58 (6.6)	50 (5.7)	32 (3.7)	17 (1.9)	11 (1.3)	6 (0.7)	582 (66.7)
Total	Age	<1	3	1	1	1	0	0	0	6 (0.7)
		1-14	50	7	15	8	4	3	6	93 (10.6)
		15-24	38	15	7	8	7	2	4	81 (9.3)
		25-44	250	52	43	18	13	7	0	383 (43.9)
		45-64	115	21	13	9	9	4	0	171 (19.6)
		≥65	105	11	12	6	1	4	0	139 (15.9)
	Grand Total		561 (64.2)	107 (12.3)	91 (10.4)	50 (5.8)	34 (3.8)	20 (2.3)	10 (1.2)	873 (100)

#### Distribution of bacterial isolates against gender, age, and specimen type

The distribution of bacterial isolates against gender, age, and specimen type is depicted in **Table 2**. Among the clinical samples collected, 175 (20.0%) were culture-positive, of which 110 (62.9%) were female and 65 (37.1%) were male. The majority of the isolates, 42.9% (75 of 175), were found in patients aged 25-44, while 22.3% (39 of 175) were found in patients aged 45–64. The majority of the isolates (56%; 98/175) were discovered in urine samples, followed by wounds (21.7%) (38/175). Out of a total of 175 bacteria, 154 (88%) were FGNB and 21 (12%) were (NFGNB)*. E. coli with* a frequency of 58.3% (102/175) and *K. pneumoniae* with a frequency of 18.3% (32/175) were the predominant FGNB, while *P. aeruginosa* 9 (5.1%) and *A. baumannii* 6 (3.4%) were the predominant NFGNB. More than three-forth, 77.5% (79/102) of *E. coli* were isolated from urine specimens, whereas 55.6% (5/9) of *P*. *aeruginosa* were from wounds.

**Table 2:** Distribution of bacterial isolates according to sex, gender type of specimens.

Gram-negative bacterial (GNB) isolates, n (%)													
Variables (number)		Fermentative GNB							Non-Fermentative GNB				
		*E. coli*	*K. pneumoniae*	*Klebsiella. Oxytoca*	*Proteus mirabilis*	*E. cloacae*	*C. koseri*	*C. barakkii*	*P aeruginosa*	*A. baumannii*	*A. lowffii*	*Sphingomonas paucimobilis*	Others
Gender	male (65)	34 (52.3)	8 (12.2)	3 (4.6)	0 (0)	2 (3.1)	1 (1.5)	1 (1.5)2	4 (6.2)	2 (3.1)	2 (3.1)	2 (3.1)	4 (5.3)
	female (110)	68 (61.8)	22 (20)	1 (0.09)	3 (2.7)	0 (0)	1 (0.1)	1 (0.09)	5 (4.5)	4 (3.6)	0 (0)	0 (0)	5 (4.5)
Age group	< 1 (3)	1 (33.3)	2 (66.7)	0 (0)	0 (0)	0 (0)	0 (0)	0 (0)	0 (0)	0 (0)	0 (0)	0 (0)	0 (0)
	1-14 (11)	7 (63.6)	2 (18.2)	0 (0)	1 (9.1)	0 (0)	0 (0)	1 (9.1)	0 (0)	0 (0)	0 (0)	0 (0)	0 (0)
	15-24 (13)	6 (46.2)	2 (15.4)	0 (0)	0 (0)	1 (7.7)	0 (0)	0 (0)	2 (15.4)	0 (0)	1 (7.7)	1 (7.7)	1 (7.7)
	25-44 (75)	40 (53.3)	14 (18.7)	3 (4)	2 (2.7)	1 (1.3)	2 (2.7)	1 (1.3)	4 (5.3)	4 (5.3)	0 (0)	1 (7.7)	3 (4.0)
	45-64 (39)	26 (66.7)	5 (12.8)	0 (0)	1 (2.6)	0 (0)	0 (0)	0 (0)	2 (5.1)	0 (0)	1 (2.6)	0 (0)	3 (7.7)
	>65 (34)	22 (62.9)	7 (20)	1 (2.9)	0 (0)	0 (0)	0 (0)	0 (0)	1 (2.9)	2 (5.7)	0 (0)	0 (0)	2 (5.7)
Types of specimens	Urine (98)	79 (80.6)	11 (11.2)	2 (2.04)	0 (0)	1 (1.0)	1 (1.0)	1 (1.02)	1 (1.02)	0 (0)	0 (0)	0 (0)	2 (2.04)
	Wound (38)	14 (36.8)	8 (21.05)	1 (2.6)	2 (5.3)	1 (2.6)	1 (2.6)	1 (2.6)	5 (13.2)	3 (7.9)	0 (0)	0 (0)	2 (5.3)
	Blood (20)	4 (20)	11 (55)	0 (0)	0 (0)	0 (0)	0 (0)	0 (0)	0 (0)	1 (5)	0 (0)	1 (5)	3 (20)
	Ear (8)	1 (12.5)	0 (0)	1 (12.5)	1 (12.5)	0 (0)	0 (0)	0 (0)	3 (37.5)	0 (0)	0 (0)	0 (0)	2 (25)
	CSF (4)	1 (25)	2 (50)	0 (0)	0 (0)	0 (0)	0 (0)	0 (0)	0 (0)	0 (0)	1 (25)	0 (0)	0 (0)
	B. Fluid (5	3 (60)	0 (0)	0 (0)	0 (0)	0 (0)	0 (0)	0 (0)	0 (0)	2 (40)	0 (0)	0 (0)	0 (0)
	Nasal (2)	0 (0)	0 (0)	0 (0)	0 (0)	0 (0)	0 (0)	0 (0)	0 (0)	0 (0)	1 (50)	1 (50)	0 (0)
Total	175 (100)	102 (58.3)	32 (18.3)	4 (2.3)	3 (1.7)	2 (1.1)	2 (1.1)	2 (1.1)	9 (5.1)	6 (3.4)	2 (1.1)	2 (1.1)	9 (5.1)

Other isolates were Providencia rettgeri, Morganella morganii, Shigella dysenteriae, Raoultella planticola, Raoultella ornithinolytica, Serratia fonticola, Burkholderia cepacia and Ralstonia pickettii.

### Antibiotic resistance profile of bacterial isolates

The antibiotic resistance profile of bacterial isolates against 20 antibiotics was presented in **Table 3**. The highest percentage level of antibiotic resistance was seen against ampicillin (86%), followed by cephalothin (73.2%) and trimethoprim/sulfamethoxazole (68.9%), whereas the lowest was recorded against meropenem (9.8%), and tobramycin (18.9%). *E. coli*, the commonest FGNB, demonstrated a higher resistance against ampicillin (81.4%) but the lowest against meropenem (4.9%). *K. pneumoniae*, the second most frequently isolated FGNB, had the highest resistance to ampicillin (100%), and the lowest to meropenem and cefoxitin (9.4% each).*P. aeruginosa*, the most frequent NFGNB, exhibited 100% resistance for ampicillin, amoxicillin-clavulanic acid, cephalothin, cefazoline, cefuroxime, cefuroxime-axetile, ceftriaxone, cefpodoxime, cefoxitin, tetracycline, trimethoprim-sulfamethoxazole, and nitrofurantoin, but the least resistance was recorded against ciprofloxacin (0%). A. baumannii, the second most commonly isolated NFGNB, demonstrated 100% resistance to ampicillin, amoxicillin-clavulanic acid, cephalothin, cefazoline, cefuroxime, cefuroxime-axetile, ceftriaxone, and cefoxitin, but lower resistance to tetracycline (16.7).

**Table 3:** Antimicrobial resistance profile of isolates.

Species (number)		Tested antibiotics, n (%)																			
		AMP	AMC	TZP	CFA	CFZ	CFU	CFX	FOX	CPD	CAZ	CRO	CFP	GM	TBM	CIP	LEV	TEC	NFT	SXT	MEM
***E. coli* (102)**	N	83	25	14	67	58	54	54	12	50	50	50	49	25	14	53	53	71	4	71	5
	%	81.4	24.5	13.7	65.7	56.9	52.9	52.9	12	49	49	49	48	25	13.7	52	52	69.6	3.9	69.6	4.9
***K.pneumoniae*(32)**	N	32	16	10	29	28	28	28	3	26	26	26	26	23	10	13	5	20	10	23	3
	%	100	50	31.3	90.6	87.5	87.5	87.5	9.4	81.3	81.3	81.3	81.3	72	31.3	40.6	15.6	62.5	31.3	72	9.4
***K. oxytoca* (4)**	N	4	2	1	3	3	3	3	1	3	3	3	3	3	3	1	1	3	1	3	0
	%	100	50	25	75	75	75	75	25	75	75	75	75	75	75	25	25	75	25	75	0
***P. aeruginosa*(9)**	N	9	9	4	9	9	9	9	9	9	4	9	3	1	1	0	2	9	9	9	3
	%	100	100	44.4	100	100	100	100	100	100	44.4	100	33.3	11	11.1	0	22.2	100	100	100	33.3
***A. baumannii*(6)**	N	6	6	4	6	6	6	6	6	6	4	4	4	2	2	4	2	1	5	4	5
	%	100	100	66.7	100	100	100	100	100	100	66.7	66.7	66.7	33	33.3	66.7	33.3	16.7	83.3	66.7	83.3
***A. lowffi* (2)**	N	0	0	0	1	1	0	0	1	0	0	0	0	0	0	0	0	0	0	0	0
	%	0	0	0	50	50	0	0	50	0	0	0	0	0	0	0	0	0	0	0	0
***P. mirabilis* (3)**	N	2	0	0	1	2	2	2	0	1	2	1	1	0	1	1	1	3	2	2	0
	%	66.7	0	0	33.3	66.7	66.7	66.7	0	33.3	66.7	33.3	33.3	0	33.3	33.3	33.3	100	66.7	66.7	0
***E. cloacae* (2)**	N	2	2	0	2	2	1	2	2	2	0	1	0	1	0	0	0	1	1	1	0
	%	100	100	0	100	100	50	100	100	100	0	50	0	50	0	0	0	50	50	50	0
***C. koseri* (2)**	N	1	0	0	1	1	1	0	0	0	0	0	0	0	0	0	0	1	0	0	0
	%	50	0	0	50	50	50	0	0	0	0	0	0	0	0	0	0	50	0	0	
***C. barakii* (2)**	N	2	1	0	2	1	1	2	2	1	0	0	0	0	0	0	0	0	0	0	0
	%	100	50	0	100	50	50	100	100	50	0	0	0	0	0	0	0	0	0	0	0
**Total**	N	141	61	33	120	110	105	106	35	98	89	94	86	55	31	72	64	109	32	113	16
	%	86	37	20	73.2	67	64	65	21	59.8	54	57.3	52.4	34	18.9	43.9	39	66.5	19.5	69	9.8

AMP, ampicillin; AMC, amox/clavulanic acid; TZP, piperacillin /tazobactam; CFA, cephalothin, CFZ, cefazoline; CFU, cefuroxime; CFXA, cefuroxime-Axetile; FOX, cefoxitin; CPD, cefpodoxime; CAZ, ceftazidime; CRO, ceftriaxone; CFP, cefepime; GM, gentamicin; TBM, tobramycin; CIP, ciprofloxacin; LEV, levofloxacin; TEC, tetracycline; NFT, nitrofurantoin; SXT, trimethoprim/sulfamethoxazole and MEM, meropenem.

### Multi-drug resistance profile of bacteria

The overall prevalence rate of MDR bacteria was 80.5% (132/164). All isolates of *A. baumannii* (6/6:100%) were MDR. Moreover, 90.6% of *K. pneumoniae*, 81.4% of *E. coli*, and 33.3% of *P*. *aeruginosa* of the isolates were MDR, respectively. About 7.3% (12/164) of the gram-negative bacteria were susceptible to all tested antibiotics (**Table 4**).

**Table 4:** Multidrug resistance level of bacteria isolated from different clinical specimen.

Isolates (number)	Level of antibiotics resistance n (%)								Total MDR isolates (≥ R3)
	R0	R1	R2	R3	R4	R5	R6	≥R7	
*E. coli* (102)	6(5.9)	8 (7.8)	5 (4.9)	10 (9.8)	8 (7.8)	14 (13.7)	14 (13.7)	37 (36.3)	83 (81.4)
*K. pneumoniae* (32)	0 (0)	1 (3.1)	2 (6.2)	2 (6.2)	0 (0)	2 (6.2)	2 (6.2)	23 (71.9)	29 (90.6)
*K. oxytoca* (4)	0 (0)	0 (0)	1 (25)	0 (0)	0 (0)	0 (0)	0 (0)	3 (75)	3 (75)
*P. aeruginosa* (9)	4 (44.4)	0 (0)	2 (22.2)	2 (22.2)	0 (0)	1 (11.1)	NA	NA	3 (33.3)
*A. baumannii* (6)	0 (0)	0 (0)	0 (0)	2 (33.3)	0 (0)	1 (16.7)	1 (16.7)	2 (33.3)	6 (100)
*A. lowffii* (2)	2 (100)	0 (0)	0 (0)	0 (0)	0 (0)	0 (0)	0 (0)	0 (0)	0(0)
*P. mirabilis* (3)	0 (0)	0 (0)	0 (0)	1 (33.3)	0 (0)	1 (33.3)	0 (0)	1 (33.3)	3(100%)
*E. cloacae* (2)	0 (0)	0 (0)	0 (0)	0 (0)	0 (0)	1 (50%)	0 (0)	1 (50%)	2(100%)
*C. koseri* (2)	0 (0)	1 (50%)	0 (0)	0 (0)	1 (50%)	0 (0)	0 (0)	0 (0)	1 (50%)
*C. barakkii* (2)	0 (0)	0 (0)	0 (0)	0 (0)	0 (0)	2(100%)	0 (0)	0 (0)	2(100%)
Total (164)	12 (7.3)	10 (6.1)	10 (6.1)	17 (10.4)	9 (5.5)	22 (13.4)	17 (10.4)	67 (40.9)	132 (80.5%)

R0: resistance to no antibiotics, R1-7: resistance to 1, 2, 3, 4, 5, 6, and 7 groups of antibiotics; ≥R3: resistance to 3 or more antibiotics from different classes. NA: not applicable (because only 5 different groups of anti-Pseudomonas antibiotics were tested during the study).

### Prevalence of ESBL-producing bacteria according to clinical samples

As shown in **Table 5**, about 49 (28%) Gram-negative bacilli were positive for ESBLase. Extended Spectrum β-lactamase production in relation to clinical samples was the highest in blood (55%;11/20), followed in wound (26.3%;10/38), and urine (23.5%;23/98). There was intra-species variation in ESBLase production, with the highest percentage recorded among *K. pneumoniae (*50%; 16/32) followed by *E. coli* (29.4%; 30/102) and the lowest production observed in *P. aeruginosa* (11.1%).

**Table 5:** Distribution of ESBLase producing Gram-negative bacteria in different isolated species and clinical specimens.

Isolated Spp	Types of samples							Total ESBLase
	Urine (n=98)	Blood (n=20)	Wound (n=38)	CSF(n=4)	Ear Swab (n=8)	Body fluids (n=5)	Nasal swab(n=2)	
*E. coli (n=102)*	18 (18.4%)	2 (10%)	7 (18.4%)	1 (25%)	0 (0)	2 (40%)	0 (0)	30 (29.4%)
*K. pneumoniae* (n=32)	4 (4.1%)	9 (45%)	2 (5.3%)	1 (25%)	0 (0)	0 (0)	0 (0)	16 (50%)
*K. oxytoca* (n=4)	1 (1%)	0 (0)	1 (2.6%)	0 (0)	0 (0)	0 (0)	0 (0)	2 (50%)
*P. aeruginosa* (n=9)	0 (0)	0 (0)	0 (0)	0 (0)	1 (12.5%)	0 (0)	0 (0)	1 (11.1%)
Total ESBL	23 (23.5%)	11 (55%)	10 (26.3%)	2 (50%)	1 (12.5%)	2 (40%)	0 (0)	49 (28%)

### Multidrug resistance level of bacteria according to clinical samples

**Figure 1:**
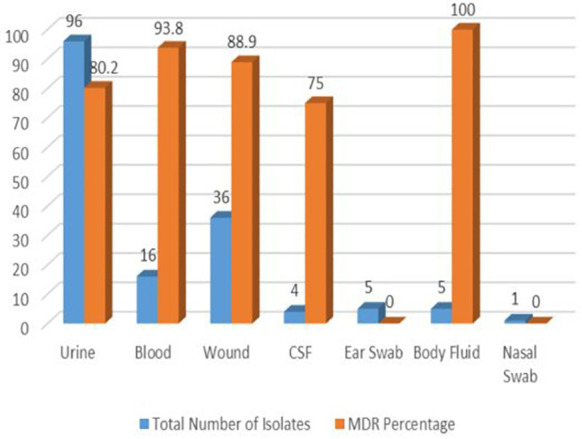
Distribution of GNB in Different Clinical Specimens.

The multidrug resistance profile of Gram-negative bacteria according to clinical samples is summarized in **Figure 1**. All bacterial isolates from body fluids (100%) were MDR, while 93.8% and 88.9% of the bacteria isolated from blood and wounds were MDR, respectively.

### Association of ESBLs production with clinical samples

Using binary logistic regression analysis, the magnitude of ESBLase production had a statistically significant association with the type of specimen. The chances of getting an ESBLase positive among bacteria isolated from blood specimens were 3.99 (95% CI = 1.47–10.80, p = 0.006) times higher than bacteria isolated from urine specimens. In multinomial logistic regression analysis, the magnitude of ESBLase production also had a statistically significant association with specimen type (P< 0.05) in which bacterial isolates from blood specimens were 3.68 (95% CI = 1.55–12.89, p = 0.007) fold higher than GNB isolated from urine specimens. However, other variables (gender and age group) did not have a statistically significant association with the magnitude of ESBLs production in our study (**Table 6**).

**Table 6:** Association of gender, age and types of specimens with magnitude of extended spectrum β-lactamases producing gram negative bacilli.

Variable (n)	ESBLs Positive n (%)	Bi-variable		Multi-variable	
		COR (CI)	P-value	OR (95% CI)	P- value
Gender					
Female (113)	31 (27.4)	Ref*	0.822	Ref*	0.999
Male (62)	18 (29)	1.08 (0.54-2.15)	0.820	1.02 (0.49, 2.13)	0.960
Age (years)					
<18 (16)	5 (45.5)	Ref*	0.773	Ref*	0.910
19-36 (66)	17 (25.8)	0.76 (0.23-2.52)	0.567	0.69 (0.20, 2.42)	0.657
37-56 (48)	15 (31.3)	1.00 (0.925-3.389)	0.927	1.06 (0.29, 3.82)	0.998
>56 (45)	12 (26.7)	0.80 (0.23-2.78)	0.988	1.00 (0.27, 3.80)	0.726
Specimen type					
Urine (98)	23 (23.5)	Ref*	0.045**	Ref*	0.048**
Blood (20)	11 (55)	3.99 (1.47, 10.80)	0.006**	3.68 (1.55, 12.89)	0.007**
Wound (38)	10 (26.3)	1.17 (0.493, 2.752)	0.728	1.10(0.48, 2.87)	0.737
Others (19)	5 (26.3)	1.17 (0.379, 3.58)	0.709	1.14 (0.39, 3.97)	0.718

** = Statistically significant association between the variables and magnitude of ESBLs producing gram negative bacteria.

Ref* =Reference.

Others = CSF (4), body fluids (5), Nasal swab (2) and ear swab (8).

COR, Crud odds ratio; AOR, Adjusted odds ratio; CI, Confidence Interval.

### Prevalence of f carbapenemase production

Out of the total 164 Gram-negative bacteria isolated, 16 isolates were resistant to meropenem. Among them 11 tested for carbapenemase production, 9 were producers and 2 were non producers. Five out of nine were metallo-lactamase producers, with the remaining four being serine carbapenemase producers. The overall prevalence of carbapenemase producing bacteria was 5.4% (9/164), since 5 *Acinetobacter* spp. were excluded from the test (**Table 7**).

**Table 7:** Distribution of carbapenemase producing gram negative bacteria against isolated species and specimen types.

Carbapenemase producer GNB				
Isolated Spp	Types of specimens			
	Wound	Urine	Body Fluid	Total
*E. coli*	0	2	1	3
*K. pneumoniae*	3	0	0	3
*P. aeruginosa*	2	1	0	3
Total	5	3	1	9

### The susceptibility of ESBLs, non-ESBLs, and MDR Gram-negative bacilli against different antibiotics tested

Generally, ESBLs producers showed higher resistance to the majority of the antibiotics tested, but non-ESBLs producers were more resistant for piperacillin/tazobactam, cefoxitin, nitrofurantoin, and meropenem. Multidrug-resistant GNB, for most of the antibiotics, showed higher resistance than non-ESBLs producers and lower than ESBLs producers (**Table 8**).

**Table 8:** Antibiotic susceptibility patterns of ESBL and carbapenemase producing gram negative bacilli.

Tested Antibiotics	ESBLs producer GNB		Carbapenemase producer GNB	
	Total No.	Susceptible, n (%)	Total No.	Susceptible, n (%)
Ampicillin	49	0 (0)	9	0 (0)
Amox/clavulanic acid	49	15 (30.6)	9	0 (0)
Piperacillin/tazobactam	49	41 (83.7)	9	1 (11.1)
Cephalothin	49	0 (0)	9	0 (0)
Cefazoline	49	0 (0)	9	0 (0)
Cefuroxime	49	0 (0)	9	0 (0)
Cefuroxime-Axetile	49	0 (0)	9	0 (0)
Cefoxitin	49	35 (71.4)	9	2 (22.2)
Cefpodoxime	49	0 (0)	9	0 (0)
Ceftazidime	49	1 (2)	9	1 (11.1)
Ceftriaxone	49	0 (0)	9	0 (0)
Cefepime	49	1 (2)	9	2 (22.2)
Gentamicin,	49	25 (51)	9	5 (55.6)
Tobramycin	49	22 (44.9)	9	3 (33.3)
Ciprofloxacin	49	20 (40.8)	9	2 (22.2)
Levofloxacin	49	28 (57.1)	9	2 (22.2)
Tetracycline	49	14 (28.6)	9	1 (11.1)
Nitrofurantoin	49	28 (57.1)	9	2 (22.2)
Trimethoprim/sulfamethoxazole	49	9 (18.4)	9	1 (11.1)
Meropenem	49	45 (91.8)	9	0 (0)

## Discussion

This study found a 20% prevalence rate of Gram-negative bacterial infection, with FGNB accounting for a significant proportion (80.0%) and NFGNB accounting for the remaining 20%. Our observation was comparable with the finding of Siou et al., 2009, who showed that nearly 15% of all GNB infections are caused by NFGNB. Contrary to our study, there was a high prevalence (54.0%) of GNB infections in Nigeria, where a significant proportion (88.9%) of them were due to MDR bacterial pathogens (Olowo-okere et al., 2020). NFGNB that were previously considered less significant pathogens have now emerged as important hospital-acquired pathogens (Malini et al., 2009). Bhargava et al., 2015 from Nepal and Sharma et al., 2014 from India demonstrated an increased prevalence rate of NFGNB of about 29.6% and 25.6%, respectively. These disparities in the prevalence of NFGNB in different healthcare situations might be due to the infection control practices and circulation of these bacterial pathogens in respective hospitals. The most concerning finding in this study was that *E. coli, K. pneumoniae, A. baumannii*, and *P. aeruginosa* were almost all of the pathogens classified as serious life-threatening pathogens (ESKAPE) by WHO, 2019. Our result concurs with the reports of many local studies (Beyene et al., 2019; Abdeta et al., 2021) and global studies (Agyepong et al., 2018; Makanjuola et al., 2018). Furthermore, cases of infections caused by infrequently isolated bacteria, including *Burkholderia cepacia, Cupriavidus pauculus, Stenotrophomonas matophilia, Methylobacterium. radiotolerns, Morayellanom. liquefaceins, and Sphingomonas paucimobilis*, have been reported for the first time in Ethiopia (Adane, 2019). In the present study also, infections involving *Raoultella ornithinolytica*, *Raoultella planticola*, and *Ralstonia pickettii* were reported as new isolates in Ethiopia. Infections with these bacteria have been reported in critically ill and immunocompromised patients with emerging opportunistic infections (Whistler et al., 2019).The difficulty in identifying these emerging pathogens in routine microbiology laboratories could be attributed to their taxonomic complexity and phenotypic similarity (Whistler et al., 2019).The isolation of this rare bacterial infection in Ethiopia in recent times may be due to the improved and highly sensitive method (VITEK Compact 2) used for bacterial identification. The machine identifies about 64 non-spore-forming Gram-negative bacteria.

The degree of drug resistance among important pathogens as depicted by this study was striking. Of the 20 antibiotics tested, the percentage of antibiotic resistance for 11 antimicrobial drugs was over 50%. The highest rates of antibiotic resistance demonstrated by the bacterial pathogens were to ampicillin, cephalothin, trimethoprim-sulfamethoxazole, and tetracycline. Comparable results were reported by many local studies (Derese et al., 2016; Gezmu et al., 2016; Terfa et al., 2018; Halaji et al, 2022; Nasrollahian et al., 2022) and in African studies (Ntirenganya et al., 2015; Agyepong et al., 2018). This practice may have evolved as a result of the antibiotics’ easy availability over the counter and the high selection pressure caused by their widespread use. Therefore, the investigation of drug-resistant pathogens like ours is extremely vital. This is not only due to its use in empirical antibiotic selection but also because antibiotic resistance surveillance has been documented as a significant method for controlling antibiotic resistance.

Specifically, *Escherichia coli*, the commonest isolate among FGNB, was less susceptible. Among the nine antibiotics in the cephalosporin category tested, the resistance rate of the bacterium ranged from 12.0% for cefoxitin to 65.7% for cephalothin, of which the resistance rate of the bacterium was >48.0% to eight drugs tested in the category. The pathogen was highly resistant to fluoroquinolones, while it was less resistant to nitrofurantoin, β-lactam/β-lactamase inhibitor combination antibiotics, carbapenem, and aminoglycosides. According to our findings, nitrofurantoin, meropenem, cefoxitin, tobramycin, and piperacillin/tazobactam were the best drugs for the treatment of infections caused by *E. coli.* Greater than 80% *K. pneumoniae*, the 2^nd^ most prevalent enterobacteria was resistance to the nine cephalosporins tested except cefoxitin. meropenem, tobramycin, nitrofurantoin, and piperacillin/tazobactam, however, were highly to moderately active drugs.

Among the isolates of NGNB, *P. aeruginosa* and *A. baumannii* were the most common isolates. Out of 20 drugs tested against *P. aeruginosa* and *A. baumannii*, 12 and 8 were found to be 100 percent resistant, respectively. The non-fermentative Gram-negative bacteria’s higher resistance could be attributed to their relative ease of acquiring plasmid-containing genes encoding Extended Spectrum β-lactamase enzymes and other resistance genes that confer resistance to many other classes of antibiotics (Agersew et al., 2013). These opportunistic pathogens are known to be intrinsically resistant to the most important classes of antibiotics. These pathogens’ higher intrinsic resistance has been linked to lower cellular permeability and higher efflux activities (Brolund, 2014).

Our findings displayed a high rate of multi-drug resistant (MDR) Gram-negative bacterial infections (80.5%). Similar studies demonstrated comparable high rates of MDR of 93.5%, 93.1%, and 87.4% in Gram-negative infection among patients in Ethiopia (Fantahun and Bayeh, 2009; Agersew et al., 2013; Eshetie et al., 2015) and 96.4% and 85.7% in Nepal (Yadav et al., 2015) and Sierra Leone (Leski et al., 2016). Discordantly, much lower MDR rates in Gram-negative bacterial infection of 74.6% by Kibret and Abera, 2011 and 68% by Moges et al., 2002 were reported in Ethiopia. Differences in the method of drug susceptibility testing, the quality and the number of antibiotics tested, the definition of MDR and misuse and overuse of antimicrobials may be sources of variation in the prevalence of antibiotic resistance seen in various studies.

Given that non-spore forming Gram-negative bacteria are heterogenous, critical assessment of MDR for each predominant species within the group seems to be more important than the percentage MDR in general. To this effect, 90.6% of *K. pneumonia* and 81.4% *E. coli* infections in our work were MDR. Our result was similar with a prevalence rate of 95.6% MDR *K. pneumonia* infection in Ethiopia (Eshetie et al., 2015) and a prevalence rate of 91.7% MDR *K. pneumonia* infection in Equatorial Guinea (Shatalov, 2015). However, a prevalence rate of 73.3% of *K. pneumoniae* MDR infection obtained in Sierra Leone (Leski et al., 2016) was significantly lower than our result. Similarly, a prevalence rate of 85.7% *E. coli* MDR infection in our study was lower than that of Ethiopia (92.9%) (Fantahun and Bayeh, 2009) and Khartoum (92.2%) (Ibrahim, 2012). Of the major NFGNB in the current study, the MDR infection rate of *A. baumannii* was 100% which was about three-fold from that of *P. aeruginosa (*33.3%). Comparable results of the MDR infection rate of *A. baumannii* with a prevalence of 95% by Mishra et al., 2013) and 96% by Shrestha et al., 2013 were reported. The prevalence of rate MDR in *A. aeruginosa* isolates in our study was comparable with that of Prakash et al., 2014 from India (31.7%).

The development and spread of ESBLase-producing non- spore-producing Gram-negative bacilli have become a major health problem globally. Although national surveillance to monitor ESBLase-producing bacteria in Ethiopia is lacking, a review of the prevalence of published data regarding ESBLase-producing bacteria in different health facilities of Ethiopia was reported to be 50% (Tafese et al., 2020). In the current study, out of 49 (28%) species of ESBLase- producing non-spore producing Gram-negative bacilli, 50% of *K. pneumoniae*, 29.4% of *E. coli*, and, 11.1% of *P. aeruginosa* were found to be ESBLase producers. Extended Spectrum β-lactamase production in relation to clinical samples was the highest in the blood (55%;11/20). Our finding was in line with the previous study in Ethiopia (Beyene et al., 2019). Blood as a major source of ESBLase-producers was significantly associated (p = 0.007).

The percentage resistance rate of imipenem (carbapenemase) against non-spore-producing Gram-negative bacteria in the current study was 9.8% which is comparably lower than previous studies conducted in Ethiopia by Legese et al., 2017 (12.1%) and Moges et al., 2019 (15.7%). However, our finding strongly disagreed with studies carried out in Tanzania (Mushi et al., 2014) where the prevalence rate of carbapenemase production was 35%. In the current study out of nine carbapenemase producers, five were Metallo-β-lactamases and four were serine carbapenemase producers.

## Conclusion

A 20% prevalence rate of Gram-negative bacterial infection with FGNB accounting for a significant proportion (80.0%) and NFGNB accounting for the remaining 20% was demonstrated. The study also found out a high prevalence rate of MDR bacteria, ESBLase, and carbapenemase, all of which is a serious concern. Antimicrobial resistance of Gram-negative bacilli should be monitored on a regular basis, and an effective infection control program should be implemented.

### Limitations of the study

The lack and/or scarcity of certain drugs such as carbapenem, tigecycline, and colistin was the major limitation of our study. Characterization of ESBLase and carbapenemase enzymes phenotypically was also another significant limitation.

## Data availability statement

The original contributions presented in the study are included in the article/supplementary material. Further inquiries can be directed to the corresponding author.

## Ethics statement

The studies involving human participants were reviewed and approved by Research Ethics Review Committee (RERC/391/19/MLS) of the department of Medical Laboratory Sciences, Addis Ababa University. Written informed consent to participate in this study was provided by the participants’ legal guardian/next of kin.

## Author contributions

YD took part in the acquisition of data, the analysis and interpretation of the data, and drafting the article. AB contributed to the article’s conception, design, and critical review. AA took part in drafting the article and critical review. All authors contributed to the article and approved the submitted version.
